# Translucency of HT lithium disilicate specimens with different thicknesses – preliminary study

**DOI:** 10.1080/07853890.2021.1897401

**Published:** 2021-09-28

**Authors:** Ma Beatriz Courela, Inês Caldeira Fernandes, Alexandra Pinto, Inês Carpinteiro, Ana Cristina Azul, Luís Proença

**Affiliations:** a Instituto Universitário Egas Moniz (IUEM), Egas Moniz Cooperativa de Ensino Superior, Caparica, Portugal; b Centro de Investigação Interdisciplinar Egas Moniz (CiiEM), Egas Moniz Cooperativa de Ensino Superior, Caparica, Portugal

## Abstract

**Introduction:**

The main advantage of using an all-ceramic system is to achieve a superior aesthetic result by increasing the translucency [1]. When dealing with darkened substrates, it is important to understand the level of translucency of the restorative materials according to their thickness, in order to adapt their application to specific clinical situations. Studies evaluating the translucency of different thicknesses of lithium disilicate, for CAD/CAM technology, are limited. This study aims to evaluate and compare the translucency of high translucency (HT) lithium disilicate with different thicknesses.

**Materials and methods:**

Lithium disilicate blocks IPS e.max® CAD HT (Ivoclar Vivadent^®^), shade A3, were sintered in a furnace (Vita Vacumat 6000 MP). The sintered blocks were cut, with a hard tissue microtome (Accutom-50; Struers Inc), in quadrangular specimens (*n* = 20). Four subgroups (*n* = 5) were defined, according to thickness (0.5, 1.0, 1.5, and 2.0 mm). The translucency of the specimens was measured using the spectrophotometer SpectroShade Micro (MHT S.p.A., Arbizzano di Negar, Italy) (*λ* = 410–680 nm). All measurements were made from 6 different areas of each specimen against a white background (*Comission Internationale de L’Eclairage* (CIE) L*=94.5, a*=–0.3, b*=–1.5) and against a black background (CIE: L*=6.1, a*=1.4, b*=–4.0), in order to obtain their L* (value coordinate), a* (red-green coordinate) and b* (yellow-blue coordinate) values under natural light (D65) [2,3]. Based on the obtained values, the translucency was calculated using the translucency parameter (TP) and the contrast ratio (CR) formulas [4]. Resulting TP and CR data were submitted to descriptive statistical analysis.

**Results:**

Mean TP values ranged from 15.5 (±0.4) to 31.6 (±2.3) and mean CR values ranged from 0.65 (±0.03) to 0.84 (±0.01). Thinner thicknesses showed higher translucency.

**Discussion and conclusions:**

Translucency is a fundamental optical property and a determinant factor when it comes to selecting the restorative material for highly aesthetical areas. This study had, therefore, the purpose of understanding which data may or may not be useful in clinical practice. The dentist should be familiar with the properties and characteristics of new and innovative materials in order to pick the one that better adapts to a certain clinical situation [3]. HT Lithium disilicate specimens showed that as thickness decreases, translucency values increase.
